# Optimal Synthesis and Application of a Si–Ti–Al Ternary Alloy as an Anode Material for Lithium-Ion Batteries

**DOI:** 10.3390/ma14226912

**Published:** 2021-11-16

**Authors:** Jaehan Lee, Young-Min Kim, Ju-Han Kim, Jee-Woon Jeong, Donghyun Lee, Jae Wook Sung, Young Ju Ahn, Jae-Hyun Shim, Sanghun Lee

**Affiliations:** 1 Carl Zeiss, Carl-Zeiss-Strasse 22, 73447 Oberkochen, Germany; jaehan.lee@zeiss.com; 2 Department of Energy Science, Sungkyunkwan University (SKKU), Suwon 16419, Korea; youngmk@skku.edu; 3 Do-Yoen Energy, Seoul 04797, Korea; j2nose@naver.com (J.-H.K.); simmany0@hanmail.net (J.W.S.); 4 Department of Advanced Materials and Energy Engineering, Dongshin University, Naju 58245, Korea; dsjjw1101@gmail.com; 5 Samsung SDI America, Auburn Hills, MI 48326, USA; dh78.lee@samsung.com; 6 Department of Mechanical and Design Engineering, Hongik University, Sejong 30016, Korea; yjahn70@hongik.ac.kr; 7 Department of Chemistry, Gachon University, Seongnam 13120, Korea

**Keywords:** lithium-ion battery, Si alloy, anode materials, real-time monitoring, lithiation

## Abstract

The development of novel anode materials for high energy density is required. Alloying Si with other metals is a promising approach to utilize the high capacity of Si. In this work, we optimized the composition of a Si–Ti–Al ternary alloy to achieve excellent electrochemical performance in terms of capacity, cyclability, and rate capability. The detailed internal structures of the alloys were characterized through their atomic compositions and diffraction patterns. The lithiation process of the alloy was monitored using real-time scanning electron microscopy, revealing that the mechanical stability of the optimized alloy was strongly enhanced compared to that of the pure silicon material.

## 1. Introduction

Graphite has become valuable as an electrode material for lithium-ion batteries (LIB) in the past several decades. Graphite exhibits many excellent properties for use in LIB, such as high specific capacity, appropriate voltage, good rate capability, good thermal stability, low volume expansion during lithiation, and excellent electronic conductivity, while its low cost is one of its biggest advantages. Nevertheless, further improvements in the energy density of LIB have been pursued to meet the demands of applications such as electric vehicles and massive energy storage systems. Towards this goal, Si has received attention as a promising substitute for graphite [1]. Although the benefits of Si-anode materials are exaggerated because of their large specific capacities, Si remains a very good candidate in terms of the stack energy density, as proposed by Obrovac and Chevrier [2]. 

However, the repeated alloying/dealloying of Li into Si leads to unwanted volume changes, which cause significant capacity losses due to material pulverization. These problems hinder the commercial use of Si anodes in LIB. To address these issues, various nanostructural modifications such as nanoparticles [3,4,5], nanowires [6,7,8], nanotubes [9,10,11], and nanosheets [12,13] have been developed for releasing large amounts of stress during cycling. The strategy of alloying Si with other elements has also been actively applied. As summarized in Obrovac and Chevrier’s review [2], either active or inactive elements can be alloyed with Si to enhance the electrochemical performance of the anode material. When nanosized Si is present in a matrix of other active elements, such as Sn, Zn, and Al, the formation of the fully lithiated phase of Si (Li_15_Si_4_) is suppressed, leading to good cycling performance. Si-Al alloy, which is cheap and highly conductive, has been used not only as an active material [14,15,16] but also as a precursor to fabricate nanostructured Si with high porosity [17,18]. Meanwhile, alloying with inactive elements, such as Fe, Ni, Co, and Ti (i.e., transition metal, TM) can reduce the overall alloy volume expansion, resulting in improved cycling performance. The inactive matrix phase buffers the volume expansion of the active Si phase and provides a continuous conduction pathway for electrons [19]. Fleischauer et al. proposed that the specific capacity of a particular Si-TM alloy can be predicted from the effective heat of formation [20]. Ti has been used as an alloy component with good electrochemical performance, and Si-Ti alloys have been manufactured using various methods [21,22,23,24]. Recently, Lee et al. reported that Ti is not simply inactive, but in fact binds to silicon atoms during lithiation [25].

Ternary alloys have also been developed to optimize the electrochemical performance of anodes. Fleischauer and Dahn investigated Si–Al–Mn alloys with over 200 compositions [26] using the combinatorial sputtering method to produce high-performance Si-alloy anodes [27,28]. However, their approach was a non-equilibrium process, which has technical limitations for practical applications on a large scale. Hence, alternative methods, such as spray-drying [29] or melt-spinning [30,31], have been proposed to improve the processability for industrial use. In particular, the melt-spinning method has been applied to other alloys for LIB anode materials [32,33]. In this work, a Si–Ti–Al ternary alloy prepared by melt-spinning was studied as an anode material for LIB. This ternary alloy system has hardly been studied as an anode material for LIB [21]. Here, a relatively uniform microstructure was obtained using arc-melting and spinning methods, which led to good electrochemical performance. To determine the optimal composition, a three-component (Si, Ti, and Al) phase diagram was used [34]. The structure of the Si alloy with good cycling ability was determined, and the suppression of the volume expansion was visually confirmed. 

## 2. Experimental Section

Si alloys for the anode were prepared by mixing Si (99.999% purity), Ti (99.9% purity), and Al (99.9% purity) in the molar ratios listed in Table 1. All alloys were prepared under an Ar atmosphere using an arc-melting process [30]. In all three compositions, the weight of Si was fixed at 50%, and only the ratios of titanium and aluminum were adjusted. The theoretical capacities of the samples, which are estimated by the lever rule using the phase diagram [35], are listed in Table 1.

**Figure 1:**
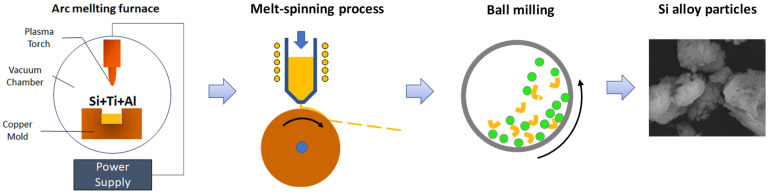
Schematic of the Si-alloy fabrication process.

The alloys were fabricated into ribbons using the melt-spinning method. The alloys were heated inside a graphite nozzle by a high-frequency induction current in an Ar atmosphere. This kept the alloys in the melt phase while they were squeezed out through a slit onto a rotating copper wheel (1400 rpm), where they were rapidly cooled. The average thickness of the alloy ribbons was ~35 µm. The cooling was very fast; consequently, the nucleation was faster than the crystal growth, resulting in very fine structures. Alloy powders were obtained by grinding 10 g of the alloy with 600 g of zirconia (5 mm diameter). A schematic of the Si-alloy fabrication process is shown in Figure 1.

The negative electrode slurry was prepared by mixing the alloy powders, Ketjen black (Lion, Tokyo, Japan), and polyimide binder (Kureha, Tokyo, Japan) with a weight ratio of 86.6:3.4:10 in N-methylpyrrolidone. The solvent was dried at 80 °C in a convection oven (JEIO TECH, Daejeon, Korea) for 2 h, and then the binder was cured at 350 °C in a hydrogen atmosphere for 2 h. Following this, 2032 coin half cells were fabricated using Li metal (anode), silicon alloy (cathode), 1 M LiPF_6_ in ethylene carbonate and diethyl carbonate with a volume ratio of 3:7 (electrolyte, Solbrain, Kongju, Korea), and a polypropylene separator (Enerever, Suwon, Korea). The electrochemical characteristics of the coin cells were analyzed in the operating voltage range of 0–2 V in constant current (CC) and constant voltage (CV) modes with rates of 0.1 C and 0.05 C, respectively. The discharging rate was 0.1 C.

The crystal structures of the silicon alloys were examined using X-ray diffraction (XRD) at ambient temperature (Rigaku MiniFlex 600, Rigaku Corporation, Tokyo, Japan) using filtered Cu-Kα radiation (λ = 1.54056 Å). The surface morphologies of the alloys were observed using field-emission scanning electron microscopy (FE-SEM; SU500, Hitachi, Tokyo, Japan). Atomic resolution images were taken using a 200 kV aberration-corrected scanning transmission electron microscope (JEM-ARM200CF, Jeol Ltd., Tokyo, Japan). The detector angle ranges for the high-angle annular dark-field (HAADF) and annular bright-field (ABF) imaging modes were 70–175 and 7.5–17 mrad, respectively. Elemental distribution maps of the samples were acquired using scanning transmission electron microscopy (STEM) along with energy dispersive X-ray spectroscopy (EDX) (JED-2300T, Jeol Ltd., Tokyo, Japan) that was equipped with a dual-type detector having a large effective solid angle (~1.2 sr). The electron probe size was ~1.1 Å. Cross-sectional transmission electron microscopy (TEM) and selected area electron diffraction (SAED) were performed. The samples were prepared using a focused ion beam (FIB, Auriga CrossBeam Workstation, Carl Zeiss, Oberkochen, Germany).

We combined a scanning electron microscope (GeminiSEM 300, Carl Zeiss, Oberkochen, Germany) with a charging circuit to visually observe the lithiation process of the alloys. One microprobe (MM3A-EM, Kleindiek, Reutlingen, Germany) was placed on a Si alloy particle inside the SEM, while the other probe was connected to the lithium metal. Then, a current of 10 µA was supplied between the probes for 1 h to lithiate the Si alloy. During lithiation, changes in the Si alloy particles and lithium metal were monitored using SEM. 

## 3. Results and Discussion

**Figure 2:**
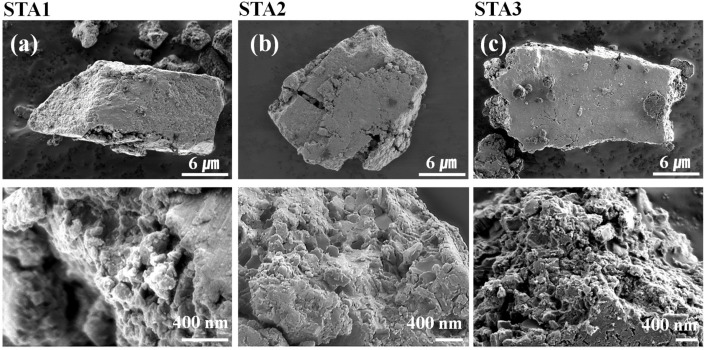
SEM images of (**a**) STA1, (**b**) STA2, and (**c**) STA3.

**Figure 3:**
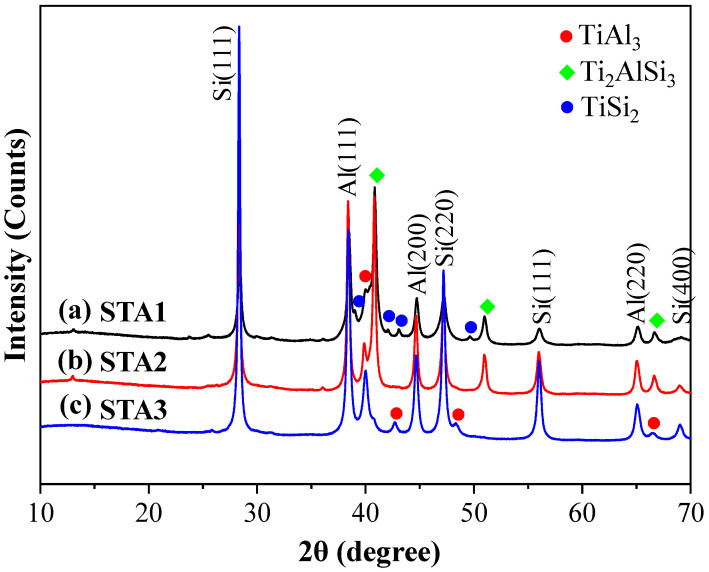
XRD patterns of (**a**) STA1, (**b**) STA2, and (**c**) STA3.

Figure 2 displays SEM images of the anode particles. All three materials consisted of irregular particles, 10–20 μm in size, with rough surfaces; there was no apparent dependence of the outer appearance on the alloy atomic ratio. The XRD patterns of the three materials are shown in Figure 3. All three materials contained Si, Al, and TiAl_3_ phases. STA1 had both TiSi_2_ and Ti_2_AlSi_3_ phases, while STA2 had only Ti_2_AlSi_3_ due to its higher Al content. However, STA3 had neither TiSi_2_ nor Ti_2_AlSi_3_, that is, a crystalline phase containing both Ti and Si was not formed in STA3.

**Figure 4:**
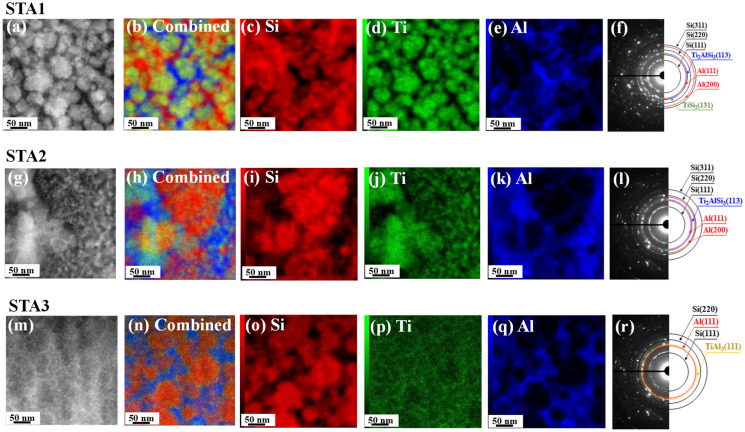
STEM images (**a**,**g**,**m**), EDX images (**b**–**e**,**h**–**k**,**n**–**q**), and SAED patterns (**f**,**l**,**r**) of STA1, STA2, and STA3.

The particle cross-sections were then investigated using STEM with EDX. As shown in Figure 4, STA1 had a significant amount of a Ti–Si composite phase (yellow color in Figure 4b), whereas STA3 had only a small amount. Ti (green color in Figure 4n) was rarely observed in STA3. In STA2, the Si-only phase (red color in Figure 4h) appeared to be richer than that in STA1, which may be related to the higher theoretical capacity of STA2. The SAED patterns (Figure 4f,l,r) also confirmed the crystalline phases of the respective materials, which were consistent with the XRD results. Figure S1 (supplementary information) shows the internal structure of STA2 as determined from STEM measurements.

**Figure 5:**
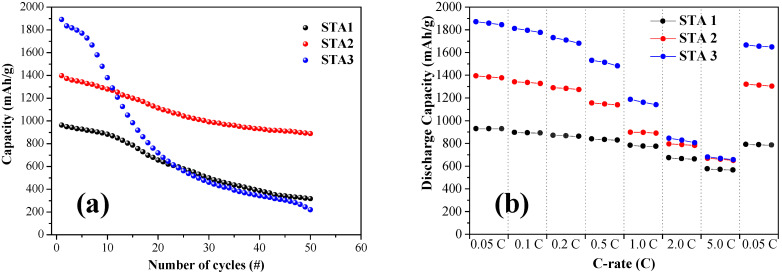
(**a**) Discharge capacity vs. cycle and (**b**) rate capability of STA1, STA2, and STA3.

Figure 5 shows the cycling performance and incremental discharge rate capability of the samples in a coin cell assembly. The cycling properties in the voltage range from 0.01 to 2.0 V were determined using a discharge rate of 0.1 C. Increasing the Al content (decreasing the Ti content) resulted in a significant increase in the initial capacity. The crystalline phases of Ti and Si, such as TiSi_2_, are not active in electrochemical lithiation [36]. Hence, STA3, which contains the most Si-only phase, had the highest capacity. However, STA3 exhibited a significant capacity decline in the early cycles owing to the lack of TiSi_2_ or Ti_2_AlSi_3_, which plays a role in charge transport as well as structural support [36]. Meanwhile, STA2, which showed an initial capacity of ~1400 mAh/g, maintained a high capacity of ~890 mAh/g after 50 cycles. The high initial capacity of STA3 at 0.05 C rapidly declined as the discharge rate increased, and at 5.0 C, it became almost the same as that of STA2. Thus, the electrochemical performance evaluation indicates that STA2 has an optimal composition as an anode material for LIB. The capacity retention and Coulombic efficiency of the samples were also consistent with this evaluation (Figure S2 in the supplementary information).

**Figure 6:**
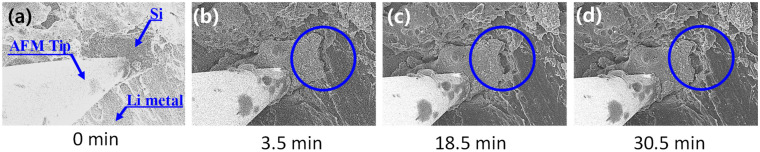
SEM images of the lithiation process in STA2. (**a**) 0 min, (**b**) 3.5 min, (**c**) 18.5 min, and (**d**) 30.5 min.

SEM combined with a charge/discharge circuit was used to visually confirm the durability of the alloy system in situ during lithiation. Figure 6 displays frames from a video of the lithiation processes in the STA2 sample. Li atoms in contact with the active material of STA2 penetrated the bulk of STA2 (circle in Figure 6b); however, no noticeable changes such as crack formation or expansion were observed in the alloy active material. Compared to pure silicon active material, where large cracks develop as lithiation progresses (Figure S3 in the supplementary information), it is apparent that STA2 had high durability throughout the lithiation process. Movies of the lithiation processes of STA2 and pure Si, from which the images in Figure 6 and Figure S3 were taken, are provided in the supplementary information.

In accordance with the above results, an effective anode material requires both Si–Ti–Al and Si-only phases for durability and capacity, respectively. When the amount of Ti is too large (STA1), the Si-only phase is not sufficiently formed; thus, the capacity of the material is reduced. On the contrary, when Ti is insufficient (STA3), the mechanical stability of the material is not guaranteed. Although this work provides a practical guideline for optimizing the composition of the Si–Ti–Al ternary alloy, there remains a future task to develop a more precise and systematic approach to achieve the optimal composition.

## 4. Conclusions

We demonstrated Si–Ti–Al ternary alloys as high-performance negative electrodes for lithium-ion batteries. The relatively uniform microstructure of the alloys was accomplished by an arc-melting and spinning process. The optimized composition for the best electrochemical performance, STA2, contains both the Ti_2_AlSi_3_ phase, which plays a role in charge transport and stability, and the Si-only phase, which is responsible for the high capacity. The excellent mechanical robustness of STA2 against lithiation during electrochemical charging was visualized in situ using SEM. No significant damage during the charging process, such as cracks or splits, was observed, which indicates a significant improvement in the mechanical characteristics of the Si anode materials.

## Figures and Tables

**Table 1:** Si, Al, and Ti molar ratios for the ternary alloys and their theoretical capacities.

Sample	Si	Ti	Al	Theoretical Capacity (mAh/g)
STA1	0.50	0.15	0.35	1111.1
STA2	0.50	0.10	0.40	1366.4
STA3	0.50	0.05	0.45	1650.6

## Data Availability

The data presented in this study are available upon request from the corresponding author.

## Supplementary Materials

The following are available online at https://www.mdpi.com/article/10.3390/ma14226912/s1, Figure S1: Atom-resolved high angle annular dark field (HAADF)-STEM images for (a) Ti2AlSi3 and (b) Si of STA2. The corresponding fast Fourier transform diffraction pattern is also shown at the right bottom of each image, Figure S2: Capacity retention and Coulombic efficiency of STA1, STA2, and STA3, Figure S3: SEM images of the lithiation process.
