# Supplementary material for: Optimal Synthesis and Application of a Si–Ti–Al Ternary Alloy as an Anode Material for Lithium-Ion Batteries
Source: Materials (Basel). 2021 Nov 16;14(22):6912. doi: 10.3390/ma14226912 (PMC8622031; doi:10.3390/ma14226912)
Supplement: Supplementary file 1 [file materials-14-06912-s001.zip › materials-1421776-supplementary.pdf]

Supplementary Information

Optimal Synthesis and Application of a Si–Ti–Al Ternary Alloy as an Anode Material for Lithium-Ion Batteries

Jae-Han Lee [1,†], Young-Min Kim [2,†], Ju-Han Kim [3,†], Jee-Woon Jeong [4], Dong-Hyun Lee [5], Jae-Wook Sung [3], Young-Ju Ahn [6], Jae-Hyun Shim [4,*] and Sang-Hun Lee [7,*]

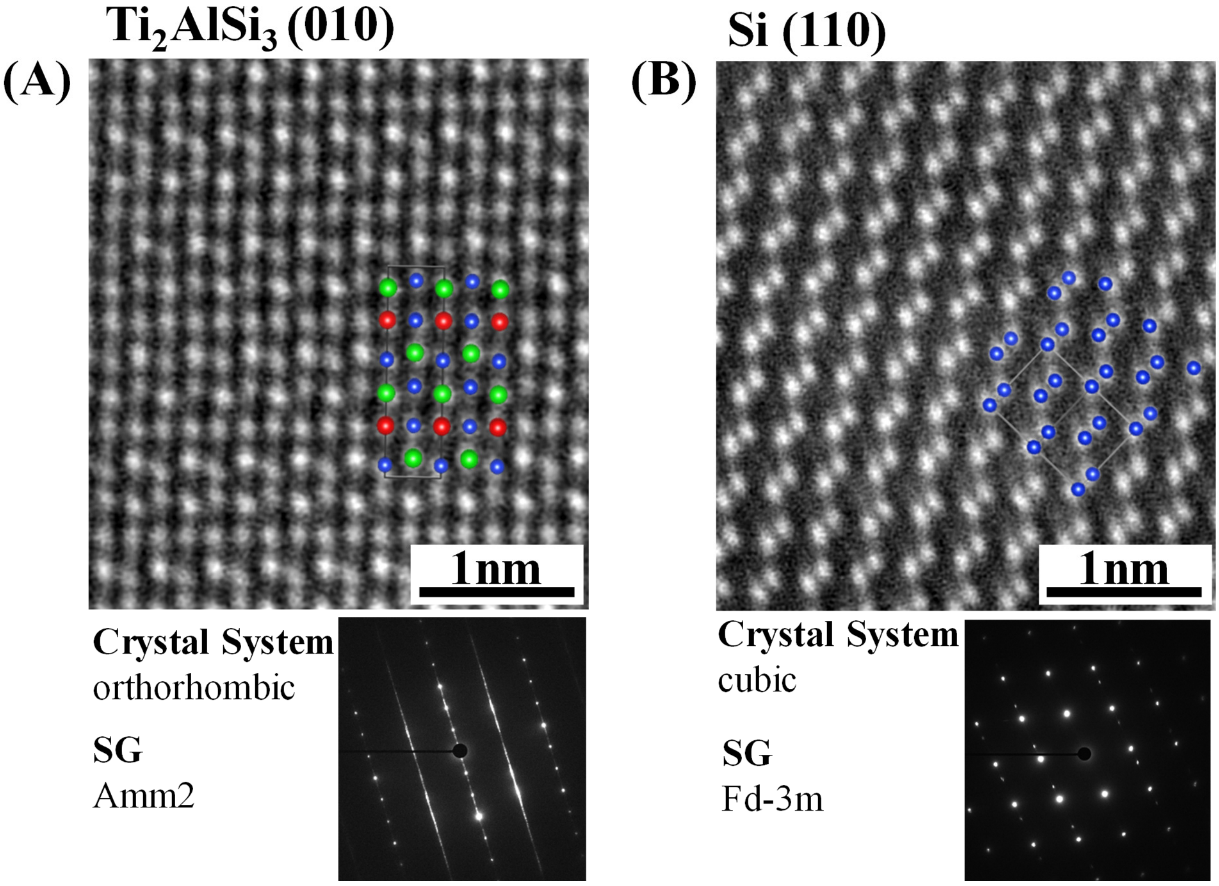

Figure S1. Atom-resolved high angle annular dark field (HAADF)-STEM images for (a) Ti_2AlSi_3 and (b) Si of STA2. The corresponding fast Fourier transform diffraction pattern is also shown at the right bottom of each image.

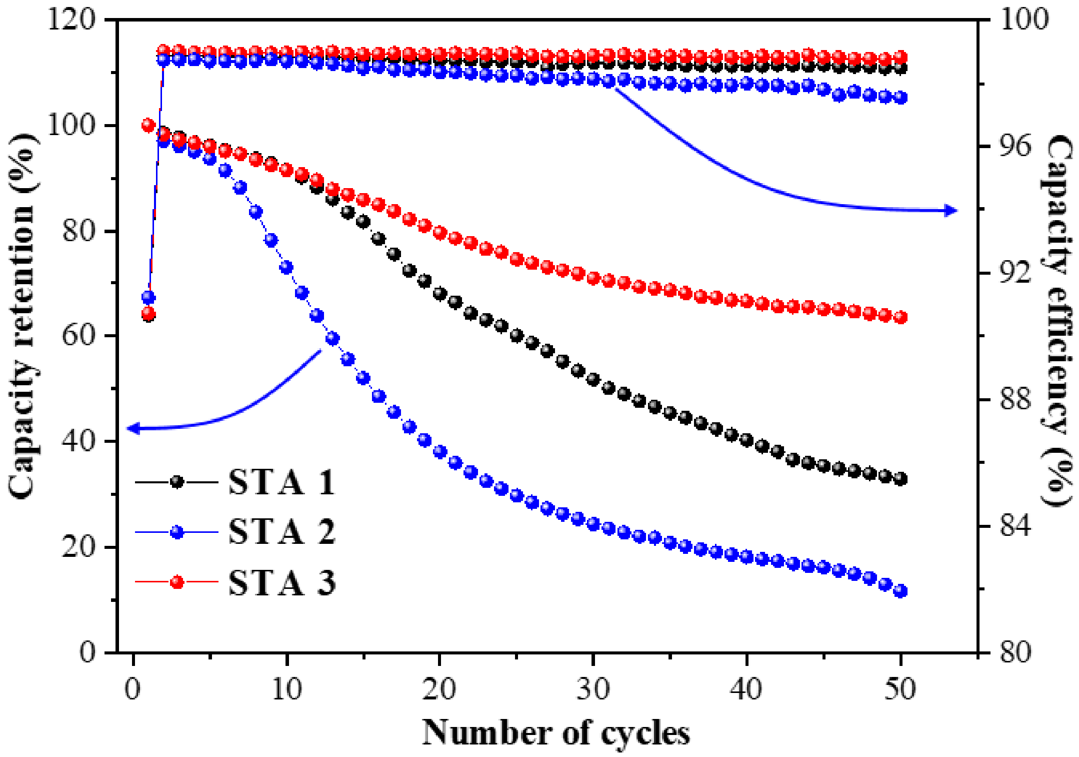

Figure S2. Capacity retention and Coulombic efficiency of STA1, STA2, and STA3.

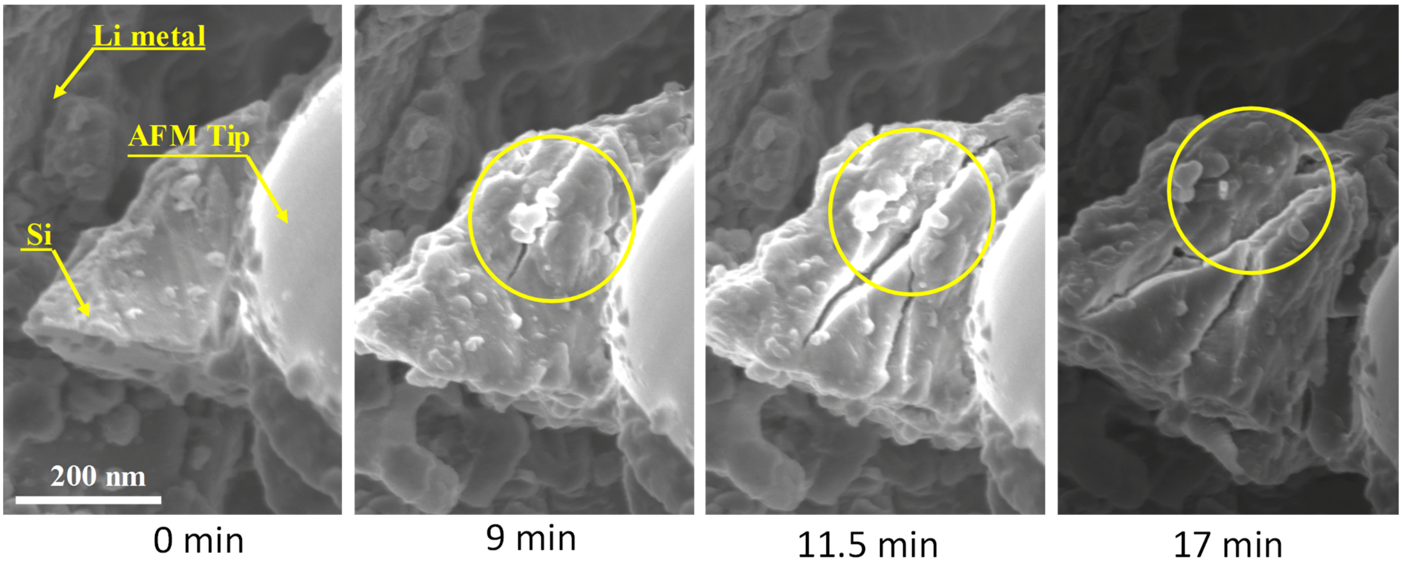

Figure S3. SEM images of the lithiation process.
